# The predictive power of hemodynamic data on postoperative neurocognitive impairment: a logistic regression and random forest approach

**DOI:** 10.1007/s13246-025-01585-3

**Published:** 2025-06-25

**Authors:** Faruk Sanberk Kiziltas, Ozhan Ozkan, Fatih Toptan, Zuhal Dogan, Kadir Gokmen, Esra Gundogdu Eryilmaz, Ibrahim Kara, Ali Fuat Erdem

**Affiliations:** 1 Department of Electrical and Electronics Engineering, Sakarya University, 54100 Sakarya, Türkiye; 2 Department of Anesthesiology and Reanimation, Sakarya Training and Research Hospital, Sakarya, 54100 Türkiye; 3 Department of Cardiovascular Surgery, Sakarya Training and Research Hospital, Sakarya, 54100 Türkiye; 4 Department of Cardiovascular Surgery, Sakarya University, Sakarya, 54050 Türkiye; 5 Department of Anesthesiology and Reanimation, Sakarya University, 54050 Sakarya, Türkiye

**Keywords:** Cardiopulmonary Bypass (CPB), Cardiopulmonary bypass graft (CABG), Logistic regression, Montreal Cognitive Assessment (MoCA) test, Neurocognitive, Partial Dependence Plot (PDP), Random Forest

## Abstract

This study assesses hemodynamic data and parameter combinations in predicting neurocognitive impairment post-cardiopulmonary bypass graft (CABG) using logistic regression and random forest algorithms. 28 patients underwent the Montreal Cognitive Assessment (MoCA) test preoperatively and one month postoperatively. Patients were grouped by MoCA score changes: Group 1 (< 2 points decrease) and Group 2 (≥ 2 points decrease). Real-time hemodynamic data were recorded during surgery, and after artifact removal, a large dataset was analyzed. Derived parameters included Absolute Maximum Decrease (AMD), areas under thresholds, and duration spent below thresholds. Logistic regression and Random Forest algorithms assessed individual and combined parameter effects. Partial Dependence Plots (PDPs) aided interpretability. Results: Logistic regression and Random Forest analyses indicated hemodynamic data have limited predictive power for neurocognitive impairment. No logistic regression analysis yielded statistically significant results, and no Random Forest model achieved high accuracy. Conclusion: Hemodynamic data alone are insufficient for prediction. Including cerebral oxygen saturation, micro emboli, and hematocrit may improve model performance. Larger sample sizes and long-term follow-up are recommended for better accuracy. This study provides a basis for future research to mitigate postoperative cognitive dysfunction.

## Introduction

The first heart-lung machine was designed by Dr. John Gibbon in 1953. The heart-lung machine he designed was used to successfully repair an atrial septal defect [1]. Advancements in surgical techniques, anesthesia, and medical device technology have improved the success rates of Cardiopulmonary Bypass (CPB) operations. However, neurocognitive impairment is significantly observed following complex and major procedures such as open-heart surgery [2–6]. The etiology of neurocognitive impairment after CPB remains unresolved and is thought to be multifactorial. Intraoperative hypotension and multiple embolisms are two primary mechanisms proposed to cause brain injury during cardiopulmonary bypass graft (CABG) operations [6, 7]. In general, there is conflicting evidence regarding the optimal Mean Arterial Pressure (MAP) value to reduce neurocognitive impairment following cardiac surgery. Numerous studies have been conducted to determine the effects of high and low blood pressure on brain injury [6, 8–11].

There are many neuropsychological tests used to evaluate neurocognitive impairment, with the Montreal Cognitive Assessment (MoCA) being one of the most commonly utilized [12]. MoCA was developed in 1995 in Montreal by Dr. Ziad Nasreddine to detect mild neurocognitive impairment in patients. The test evaluates various cognitive domains, including attention, visuospatial abilities, naming, abstraction, memory, delayed recall, orientation, and language [13].

Random Forest (RF) is widely used in biomedical and clinical prediction models due to its robustness and ability to handle high-dimensional data [14, 15]. RF models have been particularly effective in medical classification problems, making them suitable for evaluating the predictive power of intraoperative hemodynamic data on neurocognitive impairment.

To enhance model interpretability, Partial Dependence Plots (PDP) have been employed to illustrate the relationship between individual predictors and the predicted outcome [16, 17]. PDPs provide insights into how specific hemodynamic variables influence neurocognitive impairment, helping to bridge the gap between machine learning predictions and clinical decision-making.

This study was conducted to understand neurocognitive impairments, a common outcome of CABG, and to develop strategies to prevent these impairments. The aim of this study is to investigate the effectiveness of hemodynamic data recorded during surgery and the derived parameter combinations in predicting postoperative neurocognitive impairment. Specifically, the research aimed to determine whether detailed hemodynamic data recorded during the surgical process contribute to predicting neurocognitive changes. By combining statistical methods with machine learning approaches capable of capturing complex and nonlinear patterns, the study offers a more comprehensive analysis. Unlike similar studies, this research analyzed hemodynamic values and parameters derived from these values using mathematical methods not individually or collectively but as grouped combinations. Additionally, this study provides a novel perspective on the impact of hemodynamic parameters on neurocognitive outcomes, with a unique approach analyzing these parameters separately during bypass and cross-clamp periods and is the first to incorporate machine learning algorithms in this context. This approach aimed to identify which combinations of parameters have stronger or weaker collective effects. Furthermore, the study explored whether modifications in two or more parameters could positively influence neurocognitive outcomes.

## Materials and methods

The study was approved by the Ethics Committee of the Sakarya University Faculty of Medicine on February 12, 2023, with approval number 220,294 and conducted with 28 patients. All patient data used in this study were fully anonymized before analysis, with no personally identifiable information retained. The dataset contained only numerical and categorical variables relevant to the study, ensuring compliance with ethical and data protection regulations. Patients with depression, aged 80 years or older, undergoing aortic or mitral valve surgery, having diabetes, or with an ejection fraction (EF) < 50% were excluded from the study. No additional filtering or stratification was applied, and all patients meeting the criteria were included. Missing values were handled through standardized techniques, ensuring consistency across all data points. Outliers were identified using clinically relevant thresholds and excluded only if they were deemed physiologically implausible.

The recommended score for the evaluation of the MoCA test is 26 points or higher [18]. However, this cutoff value is debated in the literature due to factors such as the age, educational level, cultural background of the studied population, and screening conditions [19, 20]. Neurocognitive impairment is defined as a decrease of 2 or more points in the MoCA score [21, 22]. Recommendations regarding postoperative neurocognitive impairment emphasize the importance of performing neuropsychological assessments, such as the MoCA test, 30 days after surgery to better detect neurocognitive impairments [8].

To evaluate the presence of neurocognitive impairment, the MoCA test was administered to patients by Anesthesiology and Reanimation Specialists at Sakarya Training and Research Hospital before surgery, and by Cardiovascular Surgery Specialists one month after surgery. The Turkish version 8.1 of the MoCA test was used in this study. Based on the MoCA test conducted in the first postoperative month, patients were divided into two groups:

- Group 1 (*n* = 18): Patients with a MoCA score drop of < 2 points.
- Group 2 (*n* = 10): Patients with a MoCA score drop of ≥ 2 points.

Throughout the surgery, hemodynamic data recorded in real-time via the anesthesia device and monitoring system were analyzed to examine changes in the patient’s cognitive status and to perform classification. The recorded parameters included systolic invasive blood pressure (IBP Systolic), diastolic invasive blood pressure (IBP Diastolic), mean invasive blood pressure (IBP Mean), peripheral oxygen saturation (SpO₂), and pulse rate. Additionally, demographic information of the patients, pump duration, cross-clamp time, and the average flow rate during the pump period were also utilized in the study.

The hemodynamic data were collected using the ArcBox device, developed by Ordinatrum Healthcare Information Technologies, and transferred to the company’s information management system software.

A total of 730,870 hemodynamic data points were obtained during the surgeries of 28 patients. The hemodynamic data were recorded at an average interval of 2.4 s.

Salmasi et al. [23] removed artifacts in their study using IBP Mean data based on the following criteria:

- IBP Systolic ≤ 20 mmHg or IBP Systolic ≥ 300 mmHg.
- IBP Systolic ≤ IBP Diastolic + 5 mmHg.
- IBP Diastolic ≤ 5 mmHg or IBP Diastolic ≥ 225 mmHg.
- IBP Systolic variation within 1 min (in either direction) ≥ 80 mmHg.
- IBP Systolic variation within 2 min (in either direction) ≥ 40 mmHg.

In our study, artifacts were removed using the above criteria with the Python programming language. After artifact removal, a total of 563,185 hemodynamic data points remained for 28 patients during the surgery.

**Fig. 1:**
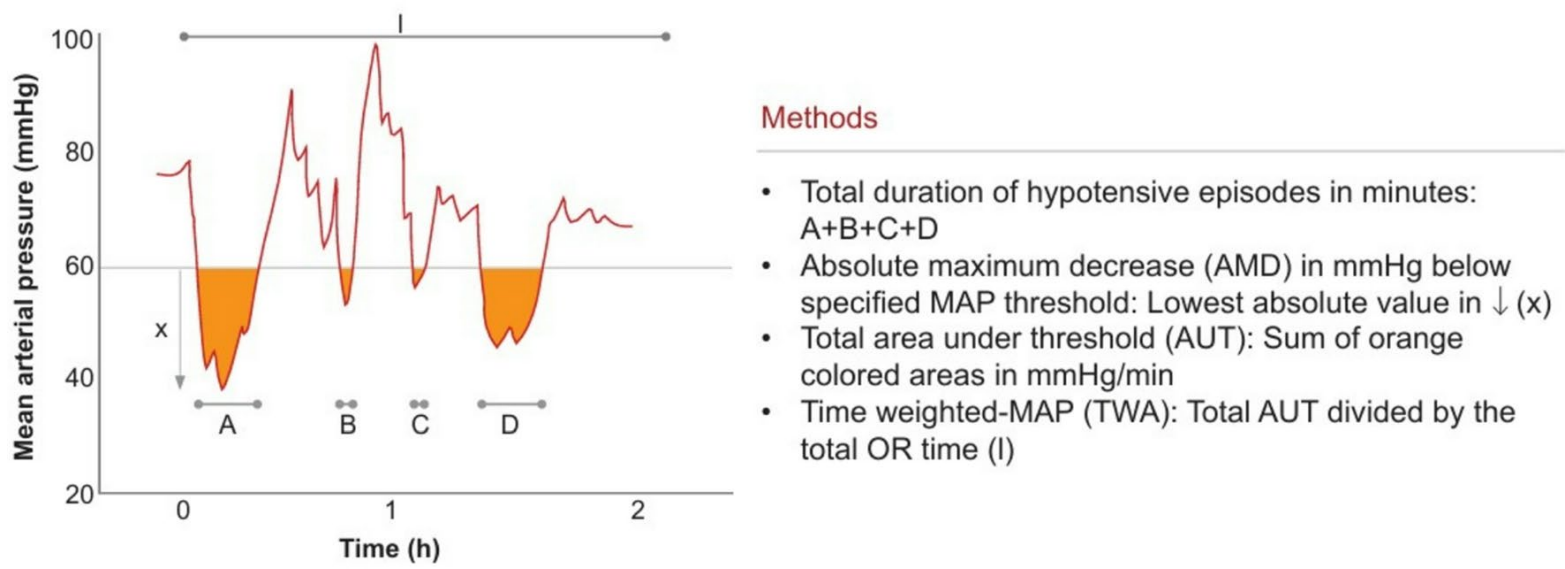
Graphical representation of the values calculated based on threshold values [24]

Gregory et al. [24], in their study, determined threshold values based on IBP Mean data and calculated the following parameters as illustrated in Fig. 1:

- Maximum drop below the specified threshold during surgery Absolute Maximum Decrease (AMD).
- Duration spent under each absolute threshold (Time_Under).
- Area under the curve between the threshold value and the recorded data over time (Area).
- Time-weighted average under each threshold (TWA_MAP).
- Cumulative time below predefined relative IBP Mean thresholds.

These metrics were used to analyze the hemodynamic impact during surgery.

In our study, three threshold values were determined for IBP Mean data: 75, 65, and 55 mmHg. Threshold values refer to MAP levels and are expressed in millimeters of mercury (mmHg). For each patient, the AMD, Time_Under, and Area parameters were calculated. As previously mentioned, the duration for each data point was assumed to be 2.4 s when calculating the Time_Under parameter, and these durations were summed.

The Area parameter was calculated using the Riemann method. The Riemann sum is a technique used to approximate the integral of a function over a specific interval. In this method, the area under a curve is divided into numerous rectangles, and the areas of these rectangles are summed. Mathematically, this method can be expressed as follows:

1 $$\:\begin{array}{c}Area=\sum\:_{i=1}^{n}\left(Threshold-IB{P}_{{Mean}_{i}}\right)*2.4\end{array}$$

For the analyses, various parameters were utilized based on the threshold values of 75, 65, and 55 mmHg during the bypass and cross-clamp periods. These parameters are as follows:

Bypass Period for Thresholds 75, 65, and 55 mmHg:

- Maximum drop below the threshold (Bypass_AMD_75, Bypass_AMD_65, Bypass_AMD_55).
- Duration spent below the threshold (Bypass_Time_Under_75, Bypass_Time_Under_65, Bypass_Time_Under_55).
- Area under the curve between the threshold and the data (Bypass_Area_75, Bypass_Area_65, Bypass_Area_55).

Cross-Clamp Period for Thresholds 75, 65, and 55 mmHg:

- Maximum drop below the threshold (Cross_AMD_75, Cross_AMD_65, Cross_AMD_55).
- Duration spent below the threshold (Cross_Time_Under_75, Cross_Time_Under_65, Cross_Time_Under_55).
- Area under the curve between the threshold and the data (Cross_Area_75, Cross_Area_65, Cross_Area_55).

Additional Data Considered in the Analyses:

- Patient’s age.
- Flow.
- BSA (Body Surface Area).
- Bypass duration (Bypass_min).
- Cross-clamp duration (Cross_min).

Average Hemodynamic Values During Bypass and Cross-Clamp Periods:

- Bypass Period: Average systolic (Bypass_Av_Sys), diastolic (Bypass_Av_Dia), pulse (Bypass_Av_PLS), and SpO₂ (Bypass_Av_SpO₂) values.
- Cross-Clamp Period: Average systolic (Cross_Av_Sys), diastolic (Cross_Av_Dia) and SpO₂ (Cross_Av_SpO₂) values.

For the analyses, combination groups were created for each threshold value. Combination groups, including parameters calculated during the bypass period along with age, flow, and BSA values, are presented in Table 1 Similarly, combination groups, including parameters calculated during the cross-clamp period along with age, flow, and BSA values, are presented in Table 2.

**Table 1:** Combination groups (bypass)

Combination Group 1	Combination Group 2	Combination Group 3
Age	Age	Age
Flow	Flow	Flow
BSA	BSA	BSA
Bypass_AMD_75	Bypass_AMD_65	Bypass_AMD_55
Bypass_Time_Under_75	Bypass_Time_Under_65	Bypass_Time_Under_55
Bypass_Area_75	Bypass_Area_65	Bypass_Area_55
Bypass_Av_Sys	Bypass_Av_Sys	Bypass_Av_Sys
Bypass_Av_Dia	Bypass_Av_Dia	Bypass_Av_Dia
Bypass_Av_PLS	Bypass_Av_PLS	Bypass_Av_PLS
Bypass_Av_SpO₂	Bypass_Av_SpO₂	Bypass_Av_SpO₂
Bypass_min	Bypass_min	Bypass_min
Cross_min	Cross_min	Cross_min

**Table 2:** Combination groups (cross– clamp)

Combination Group 1	Combination Group 2	Combination Group 3
Age	Age	Age
Flow	Flow	Flow
BSA	BSA	BSA
Cross_AMD_75	Cross_AMD_65	Cross_AMD_55
Cross_Time_Under_75	Cross_Time_Under_65	Cross_Time_Under_55
Cross_Area_75	Cross_Area_65	Cross_Area_55
Cross_Av_Sys	Cross_Av_Sys	Cross_Av_Sys
Cross_Av_Dia	Cross_Av_Dia	Cross_Av_Dia
Cross_Av_SpO₂	Cross_Av_SpO₂	Cross_Av_SpO₂
Bypass_min	Bypass_min	Bypass_min
Cross_min	Cross_min	Cross_min

A single combination group containing 12 parameters in Table 1; allows for a total of 4095 possible combinations. However, due to the presence of 9 common parameters across all combination groups, simply multiplying by 3 different threshold values would lead to redundant combinations. Accounting for these overlapping parameters, 11,263 unique analyses were conducted. Similarly, a single combination group containing 11 parameters in Table 2; allows for a total of 2047 possible combinations. With 8 common parameters across the 3 different threshold values for cross-clamp period, 5,631 unique analyses were conducted. When combining the analyses from both the bypass and cross-clamp combination groups, a total of 16,894 unique analyses were performed.

The data were evaluated using two different analysis methods: logistic regression analysis and random forest.

### Logistic regression analysis

Regression analysis is a statistical technique that examines the relationship between a dependent variable (outcome) and one or more independent variables (inputs). The goal is to model how the dependent variable can be predicted using the independent variables. Regression analysis is particularly useful for making predictions and understanding the factors that influence the dependent variable.

Logistic regression is used when the dependent variable is categorical. It is commonly applied to predict binary outcomes (e.g., patient/healthy). Mathematically, logistic regression can be expressed as:

2 $$\:\begin{array}{c}P\left(Y=1|X\right)=\frac{{e}^{{\beta\:}_{0}+{\beta\:}_{1}{X}_{1}+{\beta\:}_{2}{X}_{2}+\dots\:+{\beta\:}_{n}{X}_{n}}}{1+{e}^{{\beta\:}_{0}+{\beta\:}_{1}{X}_{1}+{\beta\:}_{2}{X}_{2}+\dots\:+{\beta\:}_{n}{X}_{n}}}\end{array}$$

$\:P(Y=1|X)$ The probability of the dependent variable being 1 (e.g., patient or positive outcome). *e*: The base of the natural logarithm, $\:{\beta\:}_{0}$ The intercept term. $\:{\beta\:}_{1}$, $\:{\beta\:}_{2}$, $\:{\beta\:}_{n}$ coefficients for the independent variables $\:{X}_{1}$, $\:{X}_{2}$,… $\:{X}_{n}$ [25].

In logistic regression analysis, the Omnibus P-Test is used to assess the overall association between all independent variables in the model and the dependent variable. The Omnibus P-Test checks whether the independent variables in the model are collectively significant in explaining the dependent variable.

Statistical significance refers to the concept that the results obtained in a study are unlikely to have occurred by random chance. It indicates support for the tested hypothesis or provides evidence that the relationship described in the hypothesis actually exists. Statistical significance is typically expressed through a p-value.

The 5% significance level (0.05) is commonly used. When the p-value < 0.05, it indicates that the results are not random with 95% confidence, supporting the hypothesis. However, a p-value below 0.05 alone is not sufficient. In such cases, alongside the Omnibus P-Test, scales expressing the model’s ability to explain the predicted probabilities are used. These include Cox & Snell’s R² and Nagelkerke’s R².

Cox & Snell’s R² measures the proportion of variance in the predicted probabilities that can be explained by the logistic regression model. However, this value does not theoretically reach 1, meaning that even if the model has perfect explanatory power, the R² value may not be equal to 1 [26].

3 $$\:\begin{array}{c}{R}_{C\&S}^{2}=1-{\left(\frac{{L}_{0}}{{L}_{M}}\right)}^{\frac{2}{n}}\end{array}$$

$\:{L}_{0}$: The log-likelihood value of the null model (a model that includes only the intercept and no independent variables). $\:{L}_{M}$: The log-likelihood value of the full model (a model that includes all independent variables). n: The number of observations.

Nagelkerke’s R² is a scaled-up version of Cox & Snell’s R², adjusted to theoretically reach a maximum value of 1, making it easier to interpret. Nagelkerke’s R² indicates the explanatory power of the model, with values ranging between 0 and 1. As the value approaches 1, it signifies that the model better explains the dependent variable [27].

4 $$\:\begin{array}{c}{R}^{2}=\frac{{R}_{C\&S}^{2}}{1-{L}_{0}^{\frac{2}{n}}}\end{array}$$

### Random forest

Machine learning is a collection of algorithms and methods developed to enable computers to identify patterns, make predictions, or perform classifications using data. Unlike traditional programming, machine learning does not rely on predefined rules; instead, example data is provided, and the computer generates a model from these data to perform similar operations on future data. In this study, classification was performed using the supervised learning method.

Random forest is an ensemble machine learning method composed of multiple decision trees. Each decision tree is trained using a random subset of the dataset and a random subset of features. In this process, each decision tree depends on a random vector (Θ_k) that is independent and identically distributed. The algorithm makes its final prediction based on the majority vote (for classification problems) or the average (for regression problems) of the predictions from all trees [14].

Random forest algorithm was used because it can capture complex and nonlinear interactions more effectively than logistic regression.

A Random Forest classifier H(x), is an ensemble consisting of K decision trees. Each tree h(x,Θ_k) is defined as follows:

5 $$\:\begin{array}{c}H\left(x\right)=\frac{1}{K}\sum\:_{k=1}^{K}h\left(x,{{\Theta\:}}_{k}\right)\end{array}$$

$\:H\left(x\right)$, The prediction made by the Random Forest classifier for the input vector x. *K*, the total number of trees in the Random Forest. $\:h(x,{{\Theta\:}}_{k})$, The prediction made by the *k*-th decision tree using the input vector *x* and the random vector $\:{{\Theta\:}}_{k}$. $\:{{\Theta\:}}_{k}$, The independently and randomly selected vectors for the *k*-th decision tree. These vectors determine the structural properties of the tree and the features to be used.

#### Partial dependence plots (PDPs)

Partial Dependence Plots (PDPs) are powerful visualization tools used to interpret complex machine learning models by showing the relationship between a subset of input features and the predicted outcome. By holding all other features constant, PDPs help reveal how changes in one or two input features affect the model’s predictions. PDPs are particularly useful for enhancing the interpretability of complex models, such as random forests or gradient-boosted trees. They provide insights into which features the model prioritizes and how these features influence the predictions. PDPs contribute to a better understanding of machine learning models and make the results more transparent. Especially for complex and “black-box” models, PDPs visually illustrate the impact of specific features on the outcomes, enabling a more detailed analysis [16].

PDPs graphically illustrate how the prediction function $\:\widehat{F}\left(x\right)$, changes with respect to input variables. Here, *x* represents all the variables on which the prediction function $\:\widehat{F}\left(x\right)$ depends. Let $\:{z}_{l}$ denote a specific subset of target variables, and $\:{z}_{\backslash\:l}$, represent the complementary subset, consisting of all remaining variables after excluding the target subset from the input variables. In this context, the prediction function $\:\widehat{F}\left(x\right)$ depends on both the $\:{z}_{l}$ and $\:{z}_{\backslash\:l}$ subsets of variables. This relationship forms the foundation for analyzing how changes in the target subset $\:{z}_{l}$ influence the predictions, while the complementary subset $\:{z}_{\backslash\:l}$ is held constant.

6 $$\:\begin{array}{c}\widehat{F}\left(x\right)=\widehat{F}\left({z}_{l},\:{z}_{\backslash\:l}\right)\end{array}$$

PDPs calculate the average of the model’s predictions for a specific feature. This average is computed over the values of all other features:

7 $$\:\begin{array}{c}{\stackrel{-}{F}}_{l}\left({z}_{l}\right)=\int\:\widehat{F}\left({z}_{l},\:{z}_{\backslash\:l}\right){p}_{\backslash\:l}\left({z}_{\backslash\:l}\right)d{z}_{\backslash\:l}\end{array}$$

$\:{p}_{\backslash\:l}\left({z}_{\backslash\:l}\right)$, is the marginal probability density of $\:{z}_{\backslash\:l}$ the complementary subset of features. $\:{\stackrel{-}{F}}_{l}\left({z}_{l}\right)$, represents the partial dependence of the model’s predictions on the target feature subset $\:{z}_{l}$. This is the core principle of PDPs: isolating the effect of a specific feature (or features) by averaging out the influence of all other features. This helps to understand how changes in the selected feature(s) affect the model’s predictions while keeping other variables constant.

## Results

Table 3 includes the parameters calculated during the bypass period as well as the patient’s demographic data. The relationships between each independent variable listed in this table were analyzed using logistic regression.

**Table 3:** Independent variables for bypass period

	Group 1	Group 2	*P*
Age	57.2 ± 11.4	58.2 ± 8.31	0.82
Flow	4609 ± 290.34	4740 ± 440.83	0.35
BSA	1.92 ± 0.13	1.97 ± 0.19	0.39
Bypass_AMD_75	49.94 ± 7	53.2 ± 7.1	0.25
Bypass_Time_Under_75	2114.13 ± 815.8	2198 ± 574.57	0.77
Bypass_Area_75	31,709 ± 13,242	34,678 ± 10,016	0.54
Bypass_AMD_65	40 ± 7	43.2 ± 7.1	0.25
Bypass_Time_Under_65	1403 ± 608.54	1496 ± 343.6	0.66
Bypass_Area_65	14,129 ± 7494	16,650 ± 7510.9	0.40
Bypass_AMD_55	29.94 ± 6.9	33.2 ± 7.1	0.25
Bypass_Time_Under_55	616 ± 404.8	756 ± 422.8	0.39
Bypass_Area_55	4711 ± 3223	5989,9 ± 3831.9	0.35
Bypass_Av_Sys	78.2 ± 9.54	75.17 ± 6.05	0.37
Bypass_Av_Dia	56.5 ± 5	56.9 ± 7.01	0.86
Bypass_Av_PLS	75.57 ± 14.1	72.2 ± 11.1	0.52
Bypass_Av_SpO₂	88.94 ± 9.81	88.53 ± 6.27	0.90
Bypass_min	114.33 ± 38.76	115.1 ± 24.2	0.96

Similarly, Table 4 was created for the data and results from the cross-clamp period. In this table, variables such as age, flow, BSA, and Bypass_min were not included because the results of the independent variable tests for these values are already provided in Table 3. Since there is no heartbeat during the cross-clamp period, the PLS value is not included in Table 4.

**Table 4:** Independent variable for cross clamp period

	Group 1	Group 2	*P*
Cross_AMD_75	42.17 ± 9.53	43 ± 9.4	0.83
Cross_Time_Under_75	464 ± 622	641 ± 646	0.48
Cross_Area_75	6131 ± 6637	7240 ± 5640	0.66
Cross_AMD_65	32.17 ± 9.53	33 ± 9.4	0.83
Cross_Time_Under_65	276.4 ± 334.8	311.5 ± 334.8	0.78
Cross_Area_65	2505.2 ± 2106.8	2861 ± 1811.9	0.66
Cross_AMD_55	22.17 ± 9.53	23 ± 9.4	0.83
Cross_Time_Under_55	102 ± 84.14	116 ± 72.53	0.65
Cross_Area_55	819 ± 696	944 ± 712	0.66
Cross_Av_Sys	71.4 ± 8.27	69 ± 11.22	0.53
Cross_Av_Dia	63.31 ± 8.84	61.34 ± 11.45	0.62
Cross_Av_SpO₂	73.7 ± 17.18	70.4 ± 13.6	0.61
Cross_min	73.11 ± 31.75	71.1 ± 17.38	0.86

When Tables 3 and 4 were examined, no statistically significant difference was found between Group 1 and Group 2 (*p* ≥ 0.05), and no parameter indicating a difference in cognitive impairment between the groups was identified. Similarly, logistic regression analysis performed to evaluate the predictive power of selected hemodynamic parameters on postoperative neurocognitive impairment revealed that none of the independent variables had a significant predictive effect (all p-values > 0.05). The odds ratio (OR) analysis showed values generally close to 1.0 for most hemodynamic parameters (e.g., AMD_75 OR = 1.301, Time_Under_75 OR = 1.000, Area_75 OR = 1.000), with 95% confidence intervals consistently including 1.0, further confirming the lack of significant associations. Moreover, factors such as high OR values and wide confidence intervals observed in variables like Age, Flow, BSA may have contributed to model instability, suggesting that these hemodynamic parameters alone may not be sufficient to reliably predict neurocognitive impairment. For this reason, simulations were conducted using 3 different combination groups created based on the threshold values, including the 12 independent variables provided in Tables 1 and 2. These simulations were analyzed using the Python programming language.

As a result of the 16,894 analyses conducted, the p-value was not less than 0.05 in any combination. Table 5 provides an example summary of the model for three combinations of bypass periods at three different threshold values (75 mmHg, 65 mmHg, and 55 mmHg) during the bypass period. For each threshold value, AMD, Time_Under, Area variables, and a constant term were included in the analysis. Examination of the p-values indicates that none of the variables were statistically significant (all *p* > 0.05). This suggests that the variables in the model have no significant effect on the outcome. The Exp(β) values represent the effect of a one-unit increase in the independent variables on the likelihood of the outcome. For instance, at the 55 mmHg threshold, a one-unit increase in AMD increases the probability of the outcome by 1.060 times (95% confidence interval: 0.930–1.207). However, the fact that all confidence intervals contain the value of 1 further confirms that these effects are not statistically significant.

**Table 5:** Logistic regression analysis results of AMD, time_under and area parameters as predictors of MoCA during bypass period at different threshold values (75 mmhg, 65 mmhg, and 55 mmHg)

Different Threshold		β	S.E.	p-value	Exp(β)	95% C.I.for EXP(B)	
						Lower	Upper
75 mmHg	AMD	,064	,064	,317	1,066	,940	1,209
	Time_Under	,000	,001	,975	1,000	,998	1,002
	Area	,000	,000	,871	1,000	1,000	1,000
	Constant	-4,242	3,399	,212	,014		
65 mmHg	AMD	,056	,064	,380	1,058	,933	1,200
	Time_Under	,000	,001	,858	1,000	,997	1,002
	Area	,000	,000	,675	1,000	1,000	1,000
	Constant	-3,194	2,751	,246	,041		
55 mmHg	AMD	,058	,067	,385	1,060	,930	1,207
	Time_Under	,000	,003	,855	1,000	,996	1,005
	Area	,000	,000	,975	1,000	,999	1,001
	Constant	-2,776	2,039	,173	,062		

Table 6 shows the Cox & Snell R² and Nagelkerke R² values obtained from the combinations examined in Table 5. The Nagelkerke R² values are higher than the Cox & Snell R² values and better reflect the explanatory power of the model. The highest Nagelkerke R² value was obtained at the 55 mm threshold (0.080), but even this value indicates that the model explains only approximately 8% of the variance in the dependent variable, which suggests a relatively low explanatory power.

**Table 6:** Cox & Snell R² and Nagelkerke R² values of the models created in Table 5

Threshold	Cox & Snell *R*²	Nagelkerke *R*²
75 mmHg	0,052	0,072
65 mmHg	0,057	0,078
55 mmHg	0,058	0,080

Classification was also performed using the Random Forest algorithm with the combination groups created in Tables I and II. For this analysis, 30% of the data was used as test data. The investigation did not identify any combination achieving over 70% accuracy. Among the 16,894 tested combinations, the highest accuracy achieved was 67%, while the lowest was 11%. Cross-validation accuracy values ranged between 27% and 71%.

Although high accuracy rates could not be achieved with the combinations, the partial dependence method was also applied, as in the Random Forest method, to enhance the interpretability of the complex model and to observe the significance of hemodynamic value ranges in the development of cognitive impairment.

The classification report for the Random Forest algorithm, focusing on one of the combinations that includes the variables Age, Bypass_Time_Under_55, Bypass_Area_55, and Bypass_AMD_55, is presented in Table 7.

**Table 7:** Independent variable for cross clamp period

	Precision	Recall	F1– Score	Support
0	0.56	1.00	0.71	5
1	0	0	0	4
Accuracy			0.55	9
Macro avg.	0.28	0.5	0.36	9
Micro avg.	0.31	0.56	0.4	9

For individuals without neurocognitive impairment (Class 0), precision was found to be 0.56, meaning that 56% of the instances predicted as Class 0 by the model were actually Class 0. Recall for Class 0 was 1.00, indicating that all actual Class 0 instances were correctly identified. For Class 1, the model failed to make accurate predictions and did not correctly identify any of the actual Class 1 instances. The model correctly classified 55% of all instances. The model performs well for Class 0, as it correctly identifies all true Class 0 instances. However, it performs poorly for Class 1, failing to detect any Class 1 instances. The overall accuracy is low (55%), which is primarily due to the misclassification of Class 1 instances.

**Fig. 2:**
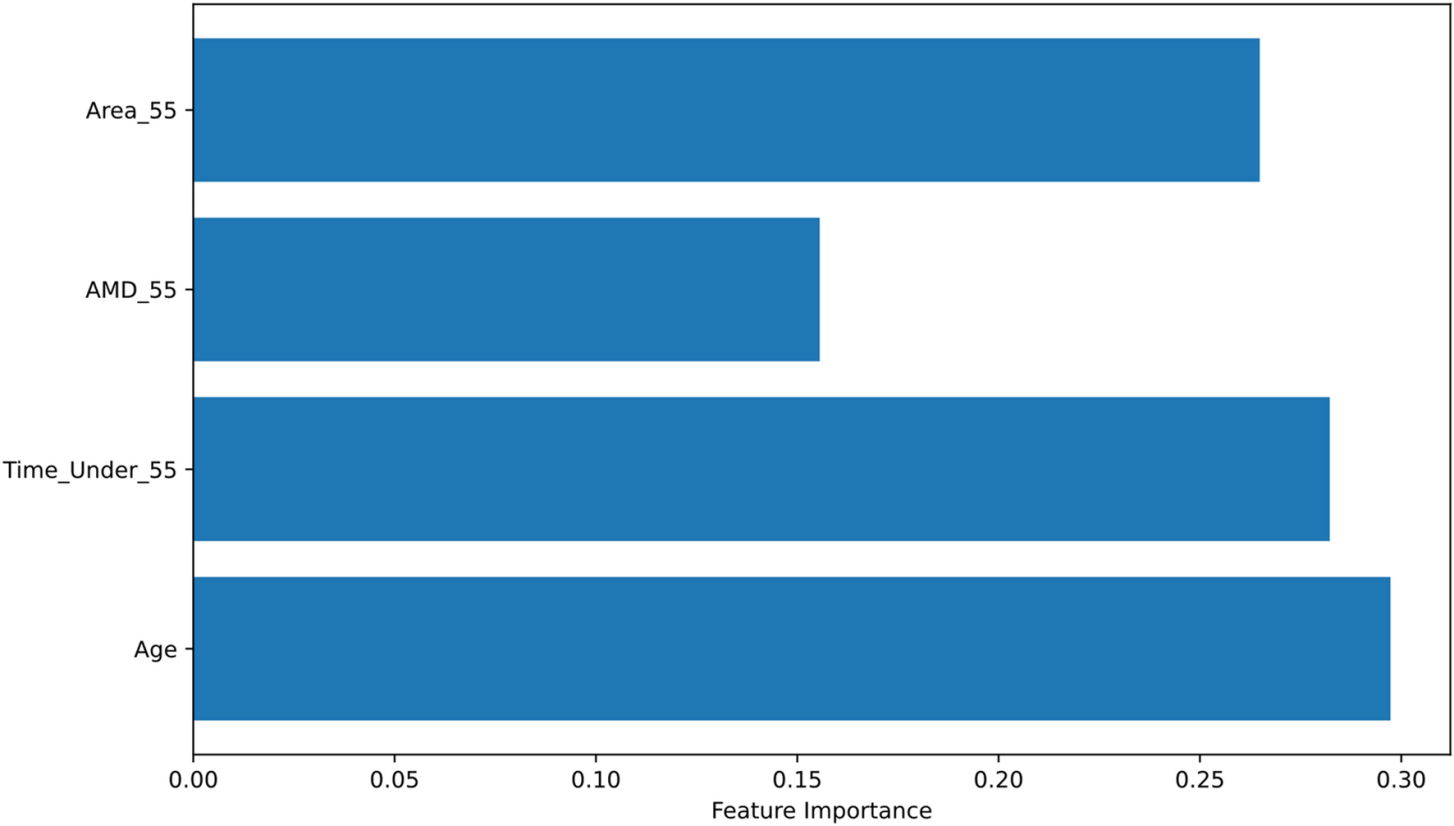
Importance of features (Age, Bypass_Time_Under_55, Bypass_Area_55, Bypass_AMD_55)

The importance ranking of the parameters for the same example model was generated (Fig. 2). It was observed that the importance of the parameters is relatively close to each other.

**Fig. 3:**
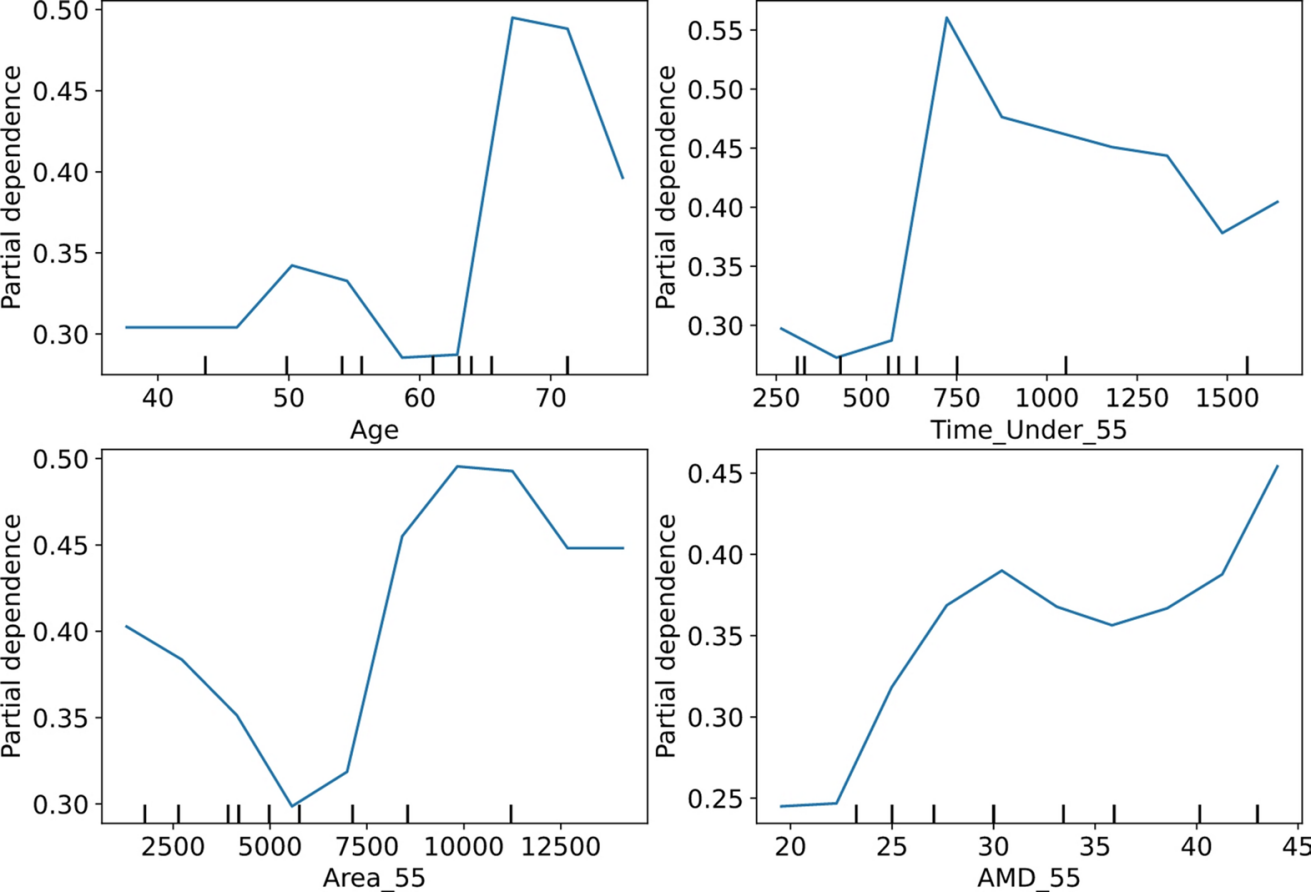
PDP of parameters (Bypass_AMD_55, Bypass_Area_55, Bypass_Time_Under_55, Age)

Figure 3 presents the Partial Dependence Plot (PDP) analyses for the selected parameters. Regarding the Age variable, the PDP graph exhibits noticeable fluctuations. While the likelihood of neurocognitive impairment increases sharply in individuals aged 60 and above, it appears to decline in individuals over 70. This fluctuation may result from complex interactions between different variables. For the Bypass_Area_55 parameter, a more irregular and fluctuating effect is observed. Initially, a significant increase is noted between 7500 and 10,000, followed by a decline beyond this point. This suggests that the area under the threshold may contribute to neurocognitive impairment up to a certain level, but its effect becomes more variable at higher values. The Bypass_Time_Under_55 parameter exhibits an increasing effect up to approximately 700, after which a decreasing trend is observed. This finding implies that a critical threshold may exist where prolonged hypotension significantly influences neurocognitive impairment. However, the interdependence between Bypass_Area_55 and Bypass_Time_Under_55 makes it challenging to draw definitive conclusions. For the Bypass_AMD_55 variable, the likelihood of neurocognitive impairment is at its lowest level around 25, but a notable increase is observed at 35 and above. This trend suggests that higher AMD values may be associated with an increased risk of neurocognitive impairment. In conclusion, among the examined parameters, Bypass_AMD_55 exhibits a clearer and more linear increasing trend, indicating a potentially stronger association with neurocognitive impairment compared to the other variables.

## Discussion

The predictive power of intraoperative hemodynamic parameters on postoperative neurocognitive impairment was evaluated using logistic regression and random forest models. Despite the extensive analysis of 30 different variables and their combinations, no statistically significant predictive relationships were identified, suggesting that intraoperative hemodynamic variables alone may not be sufficient to predict cognitive decline following CPB. In this study, hemodynamic data collected from 28 patients during surgery, along with derived parameters such as Absolute Maximum Decrease (AMD), Time Under Threshold, and Area, were comprehensively analyzed to assess their impact on neurocognitive impairment. One of the innovative aspects of this study is the separate analysis of hemodynamic parameters during the bypass and cross-clamp phases, allowing for a more detailed evaluation of how these surgical periods may influence neurocognitive outcomes. Although regression analysis and Random Forest classification indicate that hemodynamic parameters alone have a limited explanatory power, this study extends beyond existing literature by addressing these limitations and provides an important step toward a more comprehensive understanding of the relationship between hemodynamic parameters and neurocognitive impairment. These findings highlight the need for further investigation with larger datasets and additional variables to improve model performance and better elucidate the underlying mechanisms.

Newman et al. conducted a study involving 215 patients. Their study demonstrates that low MAP alone is not the primary determinant of cognitive decline. They found that a MAP below 50 mmHg led to significant impairments in cognitive functions such as spatial and figurative memory, particularly in elderly patients. Despite this, they concluded that MAP is not a major factor in cognitive decline after CPB but should be carefully monitored in elderly patients [6].

In general, there is conflicting evidence regarding the optimal MAP value to reduce neurocognitive dysfunction after cardiac surgery. Vedel et al. conducted a study on 197 patients to determine the effect of high and low blood pressure targets on brain injury. In their study, 99 patients were assigned to the 70–80 mmHg target group, and 98 patients were assigned to the 40–50 mmHg target group. Their results showed no difference in the incidence of cognitive dysfunction between the low and high MAP groups [8].

Siepe et al. studied 92 patients, dividing them into a high-pressure group (80–90 mmHg) with 44 patients and a low-pressure group (60–70 mmHg) with 48 patients. Cognitive dysfunction occurred in six patients in the low-pressure group, whereas no cognitive dysfunction was observed in the high-pressure group. The average age, cardiopulmonary bypass time, and ventilation duration were higher among the six patients who developed cognitive dysfunction. However, due to the small number of patients with cognitive dysfunction, risk factors could not be fully identified in the comparison of 86 patients. They concluded that maintaining blood pressure at 80–90 mmHg reduces cognitive dysfunction [9].

Gold et al. conducted a study with 248 patients to compare the effects of low (50–60 mmHg) and high (80–100 mmHg) blood pressure on patients. The patients were evenly divided between the groups. In the low-pressure group, the overall incidence of stroke, major cardiac events, and neurological outcomes was higher. However, no significant difference was observed between the low- and high-pressure groups in terms of cognitive dysfunction [10].

Charlson et al. investigated the effectiveness of high (80 mmHg) and custom blood pressure management strategies during CPB on morbidity, mortality, cognitive complications, and functional impairment in a study involving 412 patients. The custom pressure was determined based on the patient’s pre-bypass pressure values. They found no statistically significant difference between the high-pressure and custom-pressure groups regarding combined outcomes of mortality, cardiac, neurological, or cognitive complications, and quality-of-life impairment [11].

Pugsley et al. conducted a study involving 100 patients, suggesting that micro emboli play a role in the etiology of neuropsychological deficits after cardiopulmonary bypass. They examined the incidence of high-density micro emboli and their effects on changes in neuropsychological performance after surgery. The study concluded that the number of micro emboli during surgery has an impact on cognitive dysfunction [28].

Hu et al. investigated the potential of intraoperative cerebral tissue oxygen saturation (rSO2) to predict postoperative cognitive function in a study involving 60 patients. They found that low rSO2 could lead to postoperative cognitive decline and that optimizing rSO2 levels during surgery may be beneficial in preserving cognitive functions [29].

Mathew et al. conducted a study with 108 patients to examine the relationship between hematocrit levels and neurocognitive dysfunction after CPB. The study compared moderate hemodilution, where hematocrit levels were maintained above 27%, with deep hemodilution, where hematocrit levels were maintained between 15 and 18%, in patients aged 65 and older undergoing coronary artery bypass grafting (CABG). The effects of hemodilution on cognitive functions were significant in elderly patients. Specifically, patients in the deep hemodilution group experienced greater declines in cognitive function in the postoperative period. The study highlighted the importance of carefully monitoring hematocrit levels during CPB [30].

In recent years, machine learning (ML)-based models have played a significant role in physiological data analysis and the detection of cognitive impairment. Gao et al. developed a deep learning-based model to understand physiological responses to hemodynamic stress, transforming high-dimensional vital signs into more manageable, low-dimensional representations for analyzing physiological reactions. This approach holds promise for discovering hidden patterns in hemodynamic data [31]. Similarly, Pinto-Orellana et al. proposed a parametric data-driven model using functional near-infrared spectroscopy (fNIRS) to detect cognitive load, achieving an accuracy rate of over 86%, which enables the prediction of cognitive states based on hemodynamic variables [32]. Farajtabar et al. developed a deep neural network framework to predict arterial hemodynamic behavior in the presence of coronary artery stenosis, demonstrating that ML-based analysis of hemodynamic parameters has significant potential for the diagnosis of vascular diseases [33]. Unlike these studies, our research specifically focuses on the predictive power of intraoperative hemodynamic parameters in assessing postoperative neurocognitive impairment. While previous studies have primarily explored hemodynamic signals in the context of stress responses, cognitive load estimation, or vascular diagnostics, our study uniquely integrates hemodynamic data recorded during CABG with ML techniques to investigate their potential role in neurocognitive dysfunction.

Previous studies have emphasized the importance of various data processing techniques for biomedical signal analysis, such as frequency analysis of EMG signals and image-based bacterial detection [34, 35]. These studies demonstrate the potential of advanced data analysis techniques in healthcare applications. In a similar vein, our study applies machine learning-based classification techniques to hemodynamic data for predicting neurocognitive impairment.

Our findings indicate that hemodynamic parameters alone are insufficient to fully explain neurocognitive impairment, which aligns with previous research suggesting that hemodynamic factors are not the sole determinants of postoperative cognitive decline. Several studies have emphasized the potential contributions of cerebral oxygen saturation (rSO₂), micro emboli, and hematocrit levels in predicting cognitive outcomes following cardiac surgery. Therefore, integrating these physiological variables alongside hemodynamic data in future research may enhance the predictive accuracy of models for postoperative neurocognitive impairment, leading to a more comprehensive understanding of the underlying mechanisms.

It is particularly noteworthy that despite conducting 16,894 distinct statistical analyses, none yielded statistically significant results at the conventional α level of 0.05. In the context of multiple hypothesis testing, one would typically expect a proportion of tests to show significance purely by chance (Type I error). The complete absence of statistically significant findings across this extensive array of analyses provides compelling evidence against the notion that our results might represent statistical artifacts. This observation substantially strengthens our conclusion regarding the genuinely limited predictive capacity of hemodynamic parameters on neurocognitive impairment. Furthermore, this consistent pattern of non-significant results obviates the need for multiple comparison corrections such as Bonferroni or False Discovery Rate adjustments, which would otherwise be necessary in a scenario with numerous hypothesis tests. The robustness of these findings across all parameter combinations reinforces the validity of our methodological approach and the reliability of our conclusions.

The lack of statistical significance in the study’s results suggests that the evaluated parameters are not strong enough to predict neurocognitive dysfunction, highlighting the multifactorial nature of neurocognitive impairments. Including additional variables may increase the explanatory power of the model. Moreover, the small dataset (*n* = 28) may have contributed to overfitting or underfitting, particularly in variables like age, potentially reducing the model’s generalizability. Expanding the dataset would allow for training on a broader sample, leading to more balanced and accurate results.

Power analysis indicated that detecting a medium effect size (Cohen’s d = 0.5) with 80% power at a significance level of *p* = 0.05 would require at least 128 patients. The limited sample size in this study may have contributed to the inability to detect certain effects, increasing the risk of Type II error, meaning that some true effects may not have been identified. In single-variable analyses, the required sample size can be determined more precisely. However, in our study, multiple independent variables were used, and their interdependencies make post-hoc power estimation more challenging. Therefore, the exact number of patients required to detect significant effects could not be determined. Nevertheless, the current results were evaluated based on the available patient data, and future studies with larger sample sizes will be essential to improve the generalizability and robustness of these findings.

Long-term follow-up data can be collected to understand how postoperative neurocognitive status changes over time. Such data could enable a more comprehensive examination of cognitive changes in the postoperative period.

Given the low accuracy rates observed in our Random Forest models, a detailed SHAP (Shapley Additive Explanations) analysis was not performed, as it would not have provided additional meaningful insights. Since our classification models did not exhibit high predictive performance, the application of advanced interpretability methods such as SHAP would have been of limited utility in this context. For future research, incorporating SHAP analysis in studies with larger sample sizes and more predictive models could offer deeper insights into the relative importance of features and their interactions. This approach may help overcome the limitations of PDPs, enabling a more comprehensive interpretation of complex relationships in predictive modeling.

The study was conducted in a single-center setting with a relatively small sample size, which may affect the generalizability of the findings. Additionally, patient selection was based on predefined inclusion and exclusion criteria, which, although standardized, may introduce inherent selection bias. Future studies should aim to incorporate multicenter data from diverse populations to enhance external validity and account for potential institutional variations in surgical and anesthetic management. Expanding the dataset with larger patient cohorts will also improve statistical power, allowing for the detection of subtler patterns and more robust model performance.

Although studies have identified factors that may lead to neurocognitive impairment, it is unclear whether these significantly impact cognitive status. Our findings suggest that while both combination analyses and machine learning models have contributed to examining complex effects beyond traditional analyses, the impact of hemodynamic data on neurocognitive outcomes needs to be investigated in more detailed and comprehensive studies. Moreover, it is recommended that future research use larger patient groups and long-term follow-up data for more extensive analyses.

In conclusion, this study has shown that hemodynamic data have a limited effect on predicting neurocognitive impairment and provides an important foundation for future research. Studies conducted with additional variables could provide stronger findings for predicting and preventing neurocognitive impairments.
